# Role of microRNAs in the Regulation of Dendritic Cell Generation and Function

**DOI:** 10.3390/ijms21041319

**Published:** 2020-02-15

**Authors:** Viviana Scalavino, Marina Liso, Grazia Serino

**Affiliations:** National Institute of Gastroenterology “S. de Bellis”, Research Hospital, 70013 Castellana Grotte (Bari), Italy; vi.scalavino@gmail.com (V.S.); marinaliso@libero.it (M.L.)

**Keywords:** dendritic cells, miRNAs, inflammation

## Abstract

Dendritic cells (DCs) are antigen-presenting cells with a key role in immune responses. They act as a link between the innate and adaptive systems and they can induce and maintain immunologic tolerance. DCs are subdivided into conventional and plasmacytoid DCs. These cell subsets originate from the same bone marrow precursors and their differentiation process is determined by several extrinsic and intrinsic factors, such as cytokines, transcription factors, and miRNAs. miRNAs are small non-coding RNAs that play a crucial role in modulating physiological and pathological processes mediated by DCs. miRNA deregulation affects many inflammatory conditions and diseases. The aim of this review was to underline the importance of miRNAs in inflammatory processes mediated by DCs in physiological and pathological conditions and to highlight their potential application for future therapies.

## 1. Introduction

Dendritic cells (DCs) are a heterogenous group of antigen-presenting cells (APCs) with the ability to promote immune responses in the presence of pathogens and tissue damage signals and to induce and maintain immune tolerance [1]. DCs are the initiators and modulators of the immune response and link innate and adaptive immunity [2,3]. Furthermore, they are essential to maintain immune homeostasis and to prevent the autoimmune response to self-antigens [2].

The subset differentiation and the resulting ability of DCs to maintain homeostasis of the immune system are tightly regulated by cytokines and transcriptional factors. microRNAs (miRNAs) are an important class of post-transcriptional regulators of both innate and adaptive immunity. They play a key role in modulating both physiological and pathological processes mediated by DCs, such as hematopoietic development, cell subsets differentiation, inflammation, and autoimmunity [4,5,6,7]. The importance of miRNAs in regulating the differentiation and function of hematopoietic cells became evident when studying their relationship with the immune system and their biosynthesis. In addition, miRNAs were seen to be involved in the regulation of inflammatory pathways affecting many inflammatory conditions and diseases [8].

The aim of this review was to describe the role that miRNAs play in the regulation of DC biosynthesis and function and to also describe their involvement in inflammatory immune responses.

## 2. Dendritic Cells

The discovery of DCs is owed to Steinman and Cohn, who, in 1973, identified cells with different morphological features from mouse peripheral lymphoid organs [9]. Subsequent studies have highlighted the crucial role of DCs in the immune response [2,9,10,11].

### 2.1. Dendritic Cell Subsets

DCs can be classified as conventional (cDCs), plasmacytoid (pDCs), or monocyte-derived (mo-DCs) [12,13]. pDCs and cDCs are characterized by a high expression level of human leucocyte antigen (HLA), which corresponds to major histocompatibility complex (MHC) class II molecules [14,15].

cDCs act as sentinels in peripheral tissues, where they recognize and process multiple environmental signals and present surface antigens (Ags). Then cDCs migrate into lymph nodes (LNs) and induce CD4+ or CD8+ T-cell polarization [2,16]. cDCs are also characterized by the expression of different Toll-like receptors (TLRs), such as TLR1, TLR8, and TLR10 [14].

pDCs are a small subset of DCs that are present mainly in the blood, lymphoid tissues, and LNs. In response to pathogenic signals, pDCs produce high levels of type 1 interferons (IFN-I) and induce T helper (Th) cells response. pDCs exhibit a different pattern of surface markers from cDCs [12]. Furthermore, they express several TLRs, such as TLR1, TLR6, TLR7, TLR9, and TLR10 [14,17,18,19].

mo-DCs, also known as “inflammatory DCs” (infDCs), are DCs originating from monocyte infiltrates in lymphoid and non-lymphoid organs during inflammation or infection. In these specific conditions, they regulate innate and adaptive immune responses [20]. mo-DCs are present in peripheral tissues (intestine, lung, skin, and kidneys) and can also uptake Ags and subsequently migrate to draining LNs [21]. mo-DCs are phenotypically difficult to distinguish from cDCs because they share similar MHC-II, CD11b, and CD11c expression patterns [22].

### 2.2. Dendritic Cells Ontogeny

DCs subsets differ in terms of ontogeny, anatomical location, phenotypic morphology, and cytokines secretion patterns and functions [1,23,24].

**Figure 1:**
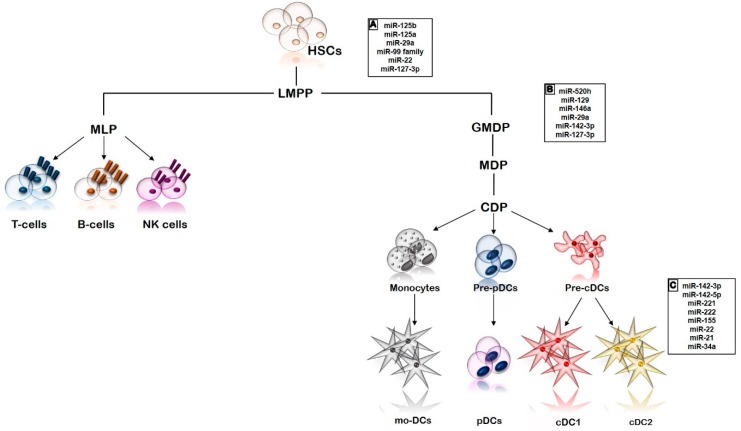
Schematic representation of the main phases of dendritic cell development and differentiation. For each step, miRNAs are involved in (**A**) maintenance and (**B**) differentiation of HSCs and (**C**) in the development and differentiation of DC subsets are reported. Abbreviations: hematopoietic stem cells (HSCs), lymphoid primed multipotent progenitor (LMPP), multipotent lymphoid progenitors (MLP), natural killer cells (NK cells), granulocyte macrophage DC progenitor (GMDP), macrophage DC progenitors (MDPs), common DC progenitors (CDPs), precursors of plasmacytoid DCs (pre-pDCs), precursors of conventional DCs (pre-cDCs), plasmacytoid DCs (pDCs), conventional DCs (cDC), and monocytes-derived DCs (mo-DCs).

As shown in Figure 1, hematopoietic stem cells (HSCs) give rise to lymphoid-primed multipotent progenitor (LMPP), which represents the precursor of the lympho-myeloid lineage. LMPP then differentiates into two distinct progenitors: MLP (multipotent lymphoid progenitor) and GMDP (granulocyte macrophage DC progenitor). T-, B- and natural killer (NK)-cells arise from MLPs, whilst GMDPs, in turn, differentiate into MDPs (macrophage DC progenitors). MDPs are proliferating cells residing in bone marrow that produce monocytes and common DC progenitors (CDPs). pre-pDCs and pre-cDCs differentiate from CDPs, then migrate into the blood and reside in an immature state in the peripheral tissues. In response to stimuli, these precursors differentiate into pDCs and cDCs subsets. Finally, mo-DCs differentiate from monocytes [14,21,25,26].

### 2.3. Dendritic Cells in Inflammation

In healthy conditions, DCs carry out many functions. They circulate in the body through the blood and can capture Ags from apoptotic cells dying during physiological turnover [27,28]. In addition, in the absence of inflammatory stimuli, DCs interact with environmental proteins and autoantigens. As a result, in order to present autoantigens to T self-reactive cells, DCs can induce immunological tolerance by deleting themselves or by stimulating the production of T regulatory (Treg) cells [29,30].

DCs also have the ability to act as sentinels in the innate immune system. In the presence of stressed or damaged tissues, such as pathogen-associated molecular patterns (PAMP) and danger-associated molecular patterns (DAMP), recognized through various pattern-recognition receptors (PRRs), they give rise to an acute inflammatory response.

Moreover, DCs act as APCs in the adaptive immune system. They recognize and process extracellular and intracellular proteins, converting them into peptides, which are then presented to T-cells by MHC molecules, thus inducing T-cell activation, together with co-stimulatory molecules and cytokines [31]. DCs are also attracted by inflammatory chemokines that are recognized by a large range of chemokine receptors (CCR1, CCR2, CCR5, and CCR6, as well as CXCR1 and CX3CR1) [32]. This process triggers the differentiation of DCs optimizing their ability as APCs.

The differentiated DCs exhibit morphological changes and express higher levels of MHC-II and other surface costimulatory molecules (CD40, CD80, and CD86) and a membrane protein associated with lysosomes (DC-LAMP), involved in Ag presentation [14]. These cells can secrete cytokines and chemokines that attract several actors of immune response in inflammatory sites and induce the differentiation of other DCs [33,34]. These differentiated DCs express CCR7 and are guided by chemokines, such as CCL19 and CCL21, to secondary lymphoid organs where T cells reside [35,36]. Therefore, DCs present Ags to the naïve CD4+ and CD8+ T cell and release cytokines, chemokines, costimulatory molecules, and proteases, initiating an immune response [14,31]. Through the secretion of distinct cytokines, such as interleukin (IL)-12, IL-23, and IL-10, DCs can stimulate the polarization of naïve T cells into Th1, Th2, Th17, or Treg cells [33].

Immune system cells, which include DCs, act as the first line in defending the host against infection and in triggering an inflammatory response. Data underline the highly important contribution of miRNAs to the development and function of immune cells [37].

## 3. microRNAs

miRNAs are small non-coding RNAs, 19–24 nucleotides long, highly conserved, and widely distributed in the genome, whose function is to recognize and regulate the gene expression of mRNA targets at the post-transcriptional level. They are able to recognize their mRNA transcript target at 3’-untranslated region (3′UTR) in a sequences-specific manner, thereby preventing or altering the production of the protein [38].

miRNA biogenesis is a complex multistep process. This process starts in the nucleus with the transcription of miRNA precursors genes (miR-gene) to primary miRNAs (pri-miRNAs) [39]. The pri-miRNAs are processed by Drosha (RNase III endonuclease) and DiGeorge syndrome critical region gene 8 (DGCR8) to create precursor-miRNAs (pre-miRNAs), which are 70-100 nucleotides long and acquire a stem-loop motif. pre-miRNAs are then exported into the cytoplasm by the exportin 5-Ran-GTP complex, forming a heterodimer that can pass through nuclear pores. pre-miRNAs are further processed by the partner proteins Dicer (second RNase III endonuclease), yielding a short double-strand miRNA. Generally, one strand of a double-strand miRNA, which represents mature miRNA, is incorporated with the RNA-induced silencing complex (RISC). This complex is composed of Argonaute proteins (AGO) and trinucleotide repeat-containing 6 proteins (TNRC6) and guides the recognition and binding of the 3’UTR of mRNA targets, thereby inducing their translational repression or degradation [8,40,41].

The primary function of miRNAs is to regulate the gene expression of the mRNA target. This mechanism has a key role in all cellular processes, including development, differentiation, metabolism, and cellular physiology [42]. In addition, miRNAs can be implicated in the pathological processes of several diseases.

### A Brief History

**Figure 2:**
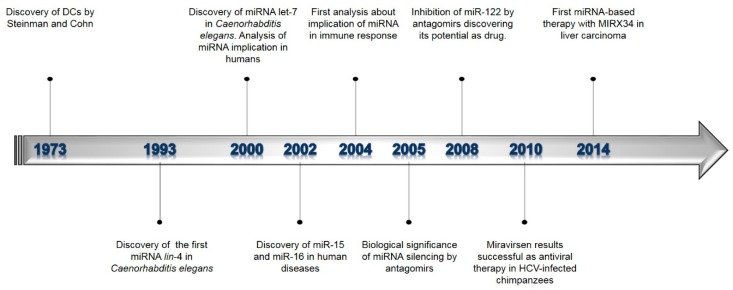
Timeline representing the main milestones of microRNAs (miRNAs) and dendritic cells (DCs) discoveries, highlighting the more significant evidence of miRNAs in immune response and therapy.

The first discovery of miRNAs dates back to 1993. Lee and colleagues detected a small RNA, namely *lin-4*, in *Caenorhabditis elegans* (Figure 2). They demonstrated that this RNA downregulated the expression of LIN-14 protein, which is involved in the larval stage progression of this nematode [43]. let-7 was the subsequent miRNA discovered, which also involved in the larval stage progression of *Caenorhabditis elegans* [44]. At first, the mechanism for regulating gene expression was thought to be exclusive to this nematode. Subsequently, this post-transcriptional regulation mechanism has been discovered in many other organisms, including humans [45]. miRNAs are evolutionarily conserved across species and can be selectively expressed in a specific tissue- or developmental-stage manner, contributing to a protein expression profile of each specific cell-type [40]. More than 2500 conserved miRNAs are present in the mammalian genome.

The first two miRNAs associated with human diseases were miR-15 and miR-16. They were identified as potential cancer genes involved in chronic lymphocytic leukemia [46]. In 2005, the biological significance of miRNA silencing was discovered through the administration of chemically engineered oligonucleotides, named “antagomirs”. These molecules were tested in vivo for the first time in mice, causing the successful silencing of miR-122 [47]. In 2008, it was demonstrated that this antagomir, later named Miravirsen, was the main candidate for the first miRNA-targeted drugs [48]. The efficacy and tolerance of this drug were reported by Landorf and co-workers a few years later. They demonstrated that the drug was able to suppress viremia in primates affected by chronic hepatitis C virus infection [49]. The first miRNA-based therapy dates back to 2014. MIRX34 is a mimic miRNA designed to replace miR-34 that results in the loss or underexpression of miR-34 in liver carcinoma [50].

## 4. microRNA in DC Development and Differentiation

miRNAs can be involved in the regulation of many aspects of innate and adaptive immune responses [51]. The involvement of miRNAs in immune response was analyzed for the first time in 2004. In their report, Chen and co-workers demonstrated that miRNAs are differentially expressed in hematopoietic lineage and can influence its differentiation [52]. This reinforced the idea that miRNAs are an integral part of networks that regulate several aspects of these immune cells. In this regard, many studies have underlined their crucial role in the development, differentiation, function, activation, and presentation of Ags by DCs, as described below (Table 1).

### 4.1. miRNAs in DCs Development

Several studies have examined the miRNA expression profile in different DC subsets and in their hematopoietic progenitors and precursors [63,70,71]. As previously described, DCs originate from HSCs in the bone marrow and, through various differentiation events driven by cytokines, progenitors, and precursors, DC subsets are produced.

Regulation of hematopoiesis is closely controlled by several extrinsic and intrinsic factors that work in synergy to determine the fate of HSCs and progenitor cells. Cytokines (GM-CSF, M-CSF, FLT3L), transcription factors, and miRNAs are the elements that determine immune cell differentiation [6,37,52,72].

One of the first analyses of the miRNA profile in HSCs dates back to 2007. To better understand the role of miRNAs in human hematopoiesis, Georgantas and collaborators determined the miRNA expression profile of human HSCs. They found 33 miRNAs expressed in CD34+ HSCs that controlled several mRNAs associated with hematopoietic differentiation [73]. Further information was obtained from an analysis in which miRNAs expressed in hematopoietic cells were compared with 98 different small RNA libraries derived from the human hematopoietic system [70]. Another group of researchers identified a set of differentially expressed miRNAs in CD34+ CD38- HSCs [53]. Among them, they selected two miRNAs, namely miR-520h and miR-129, which were increased and decreased, respectively. In particular, they demonstrated that miR-520h regulates the differentiation of the HSCs through the inhibition of ATP-binding cassette subfamily G member 2 (*ABCG2*) gene expression. Instead, miR-129 regulates eukaryotic translation initiation factor 2C, 3 (*EIF2C3*) gene, which is a key element of miRNA biogenesis, and calmodulin-binding transcription activator 1 (*CAMTA1*), which is a transcription factor involved in cell differentiation and cell cycle regulation [53].

Additional information about miRNAs related to HSCs date from 2010 onward. O’Connell and co-workers identified evolutionally conserved miRNAs, namely miR125a-5p, miR-125b-5p, miR-155, miR-130a, miR196b, miR-99a, miR-126-3p, miR-181c, miR-193b, miR-542-5p, and let7e, with a relevant function in hematopoietic cells production. The dysregulation of some of them may be correlated with hematopoietic cancers [37].

In order to determine the mechanisms involved in the differentiation of hematopoietic cell populations, several studies underlined the different roles of miR-125b based on cell context. It was shown that miR-125b was highly expressed in normal HSCs, but was downregulated in committed progenitors. With its anti-apoptotic effect, miR-125b can promote HSC survival and expansion. This antiapoptotic effect is due to a reduced mRNA expression level of two pro-apoptotic targets, Bcl2 modifying factor (*BMF*) and Krueppel-like factor 13 (*KLF13*) [54]. In addition, the overexpression of miR-125b has been associated with the onset of hematologic malignancies and can drive pathological myeloid cells expansion [37,54]. Instead, Guo S and co-workers have highlighted the central role of miR-125a in controlling the size of the stem cell population by regulating HSC apoptosis through targeting the pro-apoptotic protein BCL2 antagonist/killer 1 (*Bak1*) [55].

miR-146a regulates many aspects of HSCs, such as differentiation and survival [41,56], but also inflammatory responses [41]. It is expressed at baseline levels. Despite this, increased expression in hematopoietic cells can be induced by inflammatory stimuli [57].

HSCs are cells with a self-renewal capacity. This property is essential for their ability to maintain life-long hematopoiesis. miR-29a plays an important role during the initial phases of hematopoietic development, promoting myeloid differentiation and proliferation at the level of HSCs and regulating self-renewal. However, high levels of miR-29a have been associated with leukemogenesis [74]. In addition, in mice it has been found that DNA methyltransferase 3a (*Dnmt3a*) is a relevant target of miR-29a, indicating that miR-29a regulates the balance between HSC self-renewal and differentiation through an epigenetic mechanism [58]. miR-22 is another miRNA with the ability to regulate HSC maintenance and self-renewal via the negative regulation of ten-eleven-translocation 2 (TET2) protein levels [59].

Over the last few years, many other miRNAs related to various aspects of HSCs have been identified, including miR-142-3p and members of the miR-99 family. The former is involved in the development and differentiation of HSCs regulating the expression of interferon regulatory factor 7 (*IRF7*) [60]. Instead, members of the miR-99 family are miRNAs with a high expression level in HSCs and a key function in self-renewal [61].

Most recently, data have highlighted the importance of miR-127-3p in the self-renewal and differentiation of HSCs [62]. Crisafulli and colleagues assessed a different miRNA expression profile in Pbx1-cKO mice compared to control mice. In this model, the Pbx1 transcription factor was conditionally inactivated causing a defect in the maintenance of self-renewal. Since miR-127-3p acted as a constraint in HSC differentiation and regulation of self-renewal, this study showed that the manipulation of miR-127-3p is important to maintain correct homeostasis of the hematopoietic system [62].

### 4.2. miRNAs in DC Subsets

cDCs and pDCs, despite their common progenitors, require distinct molecular signals and different growth factors to develop and differentiate (Table 1). Indeed, miR-142-3p and miR-142-5p were differentially expressed among DC subsets and their expression was reduced in bone marrow-resident precursors, highlighting a possible involvement in regulating homeostasis [64]. miR-155 has been widely studied in DCs from several different aspects. It modulates many physiological features of the immune system, such as immune cell differentiation and development, DC functions, and surface expression markers [66,67].

Kuipers H. and colleagues have also analyzed the miRNA expression profile in pDC and cDC subsets, demonstrating that the modulation of miR-221 and miR-222 expression influences the DCs subset differentiation [63]. In another study, it was demonstrated that miR-221 can regulate DC development through the modulation of *p27kip1* [65].

Other miRNAs involved in modulating DCs subset differentiation have been identified. Among them, miR-21 and miR-34a are differentially expressed in pDCs and cDCs and play a key role in DC differentiation through the inhibition of *JAG1* and *WNT1*, two protein-coding genes required in hematopoietic differentiation and developmental processes [69]. miR-22 acts as a negative regulator of the DC transcription factor *IRF8*, thus controlling DC subset differentiation [68]. *IRF8* is a transcription factor, widely conserved in humans, that, in synergy with other transcription factors, controls the hematopoiesis in several development steps.

A study of immune cell differentiation was also conducted on human umbilical cord blood. Rajasekhar and co-workers found some miRNAs differentially expressed between myeloid progenitors and corresponding monocytes and granulocytes [71].

The importance of miRNA in DC development and differentiation is well established by the above-mentioned studies. Despite this, the expression level of a specific miRNA is crucial for the regulation of the downstream molecular mechanism. Indeed, the dysregulation of this process could give rise to hematologic cancers.

## 5. miRNAs Regulating the Role of DCs in Inflammatory Response

The relationship between miRNAs and the immune system is not only restricted to the development and the differentiation of DCs from hematopoietic precursors, but several miRNAs that closely influence the effector functions of DCs have been identified. Indeed, in the last few years, miRNAs have emerged as key regulators of inflammation, promoting or suppressing this DC-mediated process [72] (Table 2).

The innate and adaptive responses are highly regulated by cellular and molecular mechanisms through the regulation of the gene expression of specific factors in the cells involved. For this reason, the contribution of miRNAs to inflammatory processes has been amply investigated.

The miRNA expression profiles of DC progenitors under steady-state and inflammatory conditions were determined. One of the first approaches was to compare miRNA expression derived from the human hematopoietic system to miRNA expression of all other organ systems. Only five miRNAs (miR-142, miR-144, miR-150, miR-155, and miR-223) were found to be highly specific for hematopoietic cells [70]. To identify the miRNAs that regulate monocytes and DCs in steady-state and inflammatory conditions, in another study, Jansen BJ et al. analyzed their miRNA expression profiles [92]. Among the 157 screened miRNAs, 27 of these were differentially expressed between monocytes, steady-state DCs, and stimulated DCs. The analysis of the identified miRNAs in the same cell types from different donors showed that some of them were differentially expressed. This confirmed that DC development and maturation are also tightly regulated by miRNAs [92].

miR-155 has been most widely studied in the inflammatory response. Indeed, it is involved in the maturation of monocytes and bone marrow-derived dendritic cells (BMDCs) and it is required for the expression of MHC-II in differentiation mediated by GM-CSF growth factor [75]. Furthermore, miR-155 plays an essential role in controlling the intensity of the inflammatory response to microbes in human DCs [76]. It regulates the production of IL-1β, an important cytokine that stimulates LPS-mediated DC activation and modulates the TLR/IL-1 signaling pathway through *TAB2* [76]. Up-regulation of miR-155 results from exposure to inflammatory mediators, such as TNF-α and LPS [66,67], and can modulate the binding of receptors to pathogens [67].

Dueck and co-workers revealed that miR-155, under inflammatory stimuli, may regulate the expression levels of other miRNAs. In particular, in DCs, miR-99b, miR143-3p, miR-125a-5p, let-7e-5p, miR-10b-5p, and miR-210 are upregulated and miR-187-3p, miR-676-3p, miR-383-5p, miR-455, miR-672-3p, miR-181c-3p, miR-181d-5p, and miR-184-3p are repressed by miR-155. This regulation involves transcriptional activators or repressors that bind miRNA promoters and enhance or inhibit miRNA expression. One of these transcription factors is CCAAT/enhancer-binding protein β (*C/EBP-β*) [77].

In inflammatory conditions, each DC subset is specifically modulated by miRNAs. Stumpfova and colleagues evaluated the miRNA expression profiles of tolerogenic DCs (tDCs), essential to maintain immunological tolerance, and activated DCs (aDCs), after 6 h of maturation mediated by LPS and IFN-γ. They identified miRNAs that were equally expressed in both DC populations, but they also found a specific group of miRNAs upregulated in aDCs (miR-17, miR-9, miR-155, and miR-182) and another group upregulated in tDCs (miR-17, miR-133b, miR-203, and miR-23b) [93]. Another analysis of the miRNAs expression profiles in human monocytes and immature and mature DCs revealed the modulation of miR-155 and miR-221 [65]. Based on the different expression of these two miRNAs, it has been demonstrated that they can regulate various aspects of DCs, including apoptosis and IL-12 production, modulating the expression of *KPC1* and *SOCS-1*, respectively [65].

Other miRNAs expressed in most innate and adaptive immune cells belong to the miR-146 family. These miRNAs have an opposite role compared to miR-155, since miR-155 represents a pro-inflammatory element while the miR-146 family acts as an anti-inflammatory regulator. The miR-146 family consists of two evolutionarily conserved miRNAs, miR-146a and miR-146b. These miRNAs may regulate TLR signaling pathways after TLR ligand stimulation, regulating the expression of *TRAF6* and *IRAK1* [78]. Besides, through the TRAF6 adaptor molecule, miR-146a also downregulates the nuclear factor kappa-light-chain-enhancer of activated B cells (NF-kB) signaling pathway [78]. Both miRNAs may also be involved in the regulation of DC apoptosis. In fact, in another study, the role of miR-146a and miR-146b in the regulation of DC apoptosis through the miR-146a/b-TRAF6/IRAK1-NF-κB axis was identified. Specifically, it was demonstrated that miR-146a and miR-146b were negative regulators of NF-kB through the downregulation of the *TRAF6* and *IRAK1* genes [79]. Kuipers and co-workers found miR-146a and miR-146b to be differentially expressed in cDCs compared to DCs stimulated with GM-CSF in vitro (GM-CSF DCs). From these analyses, a higher expression of miR-146b, but lower expression of miR-146a, resulted in GM-CSF DC compared to cDC. These data suggested a miR-146b-mediated decrease in TLR signaling sensitivity in GM-CSF DC that might be correlated with the pro-inflammatory nature of GM-CSF [63].

miR-223 is another widely studied miRNA. It has been shown that this miRNA interacts and regulates different key elements of the inflammatory response. miR-223 is essential in innate immune responses because it regulates myeloid differentiation and granulocyte functions [94,95].

miR-22 is a miRNA that is highly conserved in vertebrate evolution and it is ubiquitously expressed in various tissues [80]. *p38*, a target of miR-22, is an important member of the MAPK family and, by regulating the activities of some interleukins, such as IL-6, it controls the process of tumor generation. In DCs, *p38* has a crucial role in DC maturation and miR-22 negatively influences this factor, reducing its translation and also reducing IL-6 expression [80].

miR-21 and miR-34a are two other miRNAs differentially expressed in pDCs and cDCs. miR-21 acts as a negative regulator of inflammation. In fact, the upregulation of this miRNA leads to low secretion of IL-6 and increased production of IL-10, underlining its anti-inflammatory effect [81]. Recently, the lack of miR-21 was associated with the increased production of inflammatory cytokines by macrophages in cardiac tissue. In the presence of an inflammatory environment, DCs express TLRs, so they can recognize DAMPs and produce inflammatory cytokines [82]. miR-34a is involved in the homeostatic control of CD1c+ DCs activation. miR-34a is also expressed in inflammatory DCs. During maturation of inflammatory DCs with TLR ligands, miR-34a is rapidly downregulated, therefore its epigenetic target, *AXL*, comes into play, leading to the interruption of DC activation, ending the inflammatory response [83]. The situation is reversed in patients affected by rheumatoid arthritis, where miR-34 is up-regulated in DC1c+ DCs. This event results in a decrease of *AXL* [83].

The miR-29 family is associated with atherosclerosis autoimmune disease, showing a crucial role of miR-29a in pro-inflammatory cytokine secretion [96]. DCs also play a key role in the regulation of tumor immunity. miR-29b was identified in tumor-associated DCs. In this context, miR-29b proved to be upregulated in normal mature DCs and significantly downregulated in tumor-associated DCs [97]. miR-29a and miR-142-3p play a key role in promoting granulopoiesis and monopoiesis and their abnormally decreased expression is responsible for a blockade of blast cell differentiation in some acute myeloid leukemia patients [98]. However, as mentioned above, the up-regulation of miR-29a is also associated with leukemia [74].

miR-142-3p has a pro-inflammatory function in mo-DCs. This was confirmed by miRNA expression analysis in the pathogenesis of systemic lupus erythematosus (SLE). In fact, the overexpression of miR-142-3p in mo-DCs and cytokines, such as CCL2, CCL5, CXCL8, IL-6, and TNF-α, result in increases in the inflammatory context [85]. Moreover, miR-142-3p, together with miR-24 and miR-30b, can regulate the activation of adaptive immune responses guided by APCs [86,87].

These findings further clarify that a finely-tuned regulation of gene expression by miRNAs is crucial for normal homeostasis and physiology since an abnormal miRNA expression could trigger the onset of malignant diseases.

## 6. miRNAs in DC-Mediated Intestinal Diseases

In the last years, many studies have evaluated the role of miRNAs in the regulation of intestinal innate immunity and their correlation with intestinal inflammatory disorders, such as inflammatory bowel disease (IBD) and celiac disease.

Multiple findings indicate that abnormal miRNA expression can condition the function of various immune cells and, therefore, can regulate the inflammatory response in IBD and colon-rectal cancer (CRC) [99]. To date, few works have been performed to study the alteration of miRNA expression in intestinal DC functions (Table 2).

The microbiota has come to be increasing of interest given its possible involvement in intestinal inflammatory diseases. In addition, it may contribute to the alteration of miRNA expression in DCs. miR-10a is expressed in intestinal DCs and, in particular, is downregulated in the inflamed mucosa of IBD patients. The abnormal expression of this miRNA in human DCs is due to the microbiota and to cytokines, such as TNF and INF-γ. This leads to the inhibition of nucleotide-binding oligomerization domain-containing protein 2 (NOD2) expression and production of IL-12 and IL-23 [88]. Xue and colleagues also demonstrated that the microbiota negatively regulates miR-10a expression in the mucosal DCs of mice with colitis. The microbiota downregulates the expression of miR-10a in intestinal DCs through TLR-TLR ligand interaction with the resulting inhibition of IL-12/IL-23p40 DC production. This highlights the importance of miR-10a in the maintenance of intestinal homeostasis [89].

In addition, it has been demonstrated that the microbiota, as well as pro-inflammatory cytokines, downregulate the expression of miR-107 in intestinal cDCs. In particular, the microbiota downregulates miR-107 through TLR-TLR ligand interaction, which leads to an increased expression of IL-23p19 and the production of IL-23. Even in this case, miR-107 may also contribute to the maintenance of intestinal homeostasis and influence the immune response to the microbiota [90].

miR-223 has a crucial role in the maintenance of homeostasis of the intestinal environment. The role of miR-223 has been investigated by Zhou and colleagues in an miR-223-deficient mouse model. In this model, a strong pro-inflammatory phenotype of DCs was found. This was supported by the production of TNF-α, IL-6, and IL-12 pro-inflammatory cytokines. Furthermore, they showed that the expression of pro-inflammatory cytokines is positively regulated by *C/EBP*-β and is a direct target of miR-223 [91]. Moreover, in bone marrow-derived DCs and intestinal DCs, the regulation of *C/EBP*-β by miR-369-3p suppresses the chronic inflammatory response [100].

NOD2 is a protein that plays an important role in immune response and inflammation. It can recognize pathogen molecules and can stimulate the immune response, stimulating the production of pro-inflammatory cytokines by DCs. NOD2 guides miR-29 expression in DCs. In a study conducted by Brain and colleagues, it was reported that the expression of miR-29 in DCs is upregulated in response to NOD2 signals. Consequently, miR-29 downregulates IL-23 by targeting IL-12p40 directly and IL-23p19 indirectly via a reduction of activating transcription factor 2 (*ATF2*). This led to the increased production of IL-23 in the intestinal mucosa of miR-29-deficient mice with colitis. Furthermore, a defect in NOD2 in the intestinal DCs of Crohn’s disease patients has been observed. The loss of this immunoregulatory pathway in intestinal DCs caused the massive production of IL-23 in response to microbes in Crohn’s disease patients. Therefore, the authors stressed that a loss of miR-29-mediated immunoregulation in DCs influences the elevated release of IL-23 in Crohn’s disease [84].

## 7. Conclusions and Perspectives

Inflammation is a complex process with many components able to promote its regulation. The wide range of reported studies emphasize the view that miRNAs control many aspects of inflammatory processes at multiple levels.

In this review, we pointed out that different miRNAs regulate many physiological aspects of DCs, including biogenesis, differentiation, maturation, and function (Figure 1). These miRNAs also have a key role in several pathological processes mediated by DCs. The abnormal expression of miRNAs that can affect DC development and differentiation, as well as DC function in inflammatory and autoimmune diseases, has been closely investigated.

Clarifying the molecular mechanisms of miRNAs in inflammatory diseases mediated by DCs may guide the production of future therapies. miRNA-based therapies may be realized through the manipulation of endogenous miRNA levels by administering miRNA inhibitors or miRNA mimics able to change the expression of target genes. Few clinical trials on miRNA-based therapies have been reported in the last decades. The main trials concern cancer-related pathologies where intratumoral injections of miRNA drugs enhance specificity and efficacy and minimize side effects. However, since Miravirsen, no clinical trial based on miRNA drugs has reached phase III.

To date, no miRNA-based therapy has been developed to treat inflammatory diseases in human clinical trials. Despite this, the use of miRNAs as therapeutic agents is very promising since a single miRNA is able to regulate the expression of numerous genes working in coordination to regulate a biological process. This characteristic makes them suitable for the treatment of complex diseases, like inflammatory diseases, that are generally associated with alterations of different pathways. However, the administration of miRNA inhibitors or miRNA mimics as drugs has been little investigated and future studies are needed to determine the effects of such treatments and their safety and toxicity as well as the route of administration.

## Figures and Tables

**Table 1:** miRNAs involved in DC development and differentiation.

	miRNA	Gene Target	Function	References
DCs development	miR-520h	ABCG2	Differentiation	[53]
	miR-129	EIF2C3 CAMTA1	Differentiation	[53]
	miR-125b	BMF, KLF13	Survival and destination	[54]
	miR-125a	Bak1	Control of stem cell size	[55]
	miR-146a		Differentiation, survival, and inflammatory response	[41,56,57]
	miR-29a	Dnmt3a	Hematopoietic development, myeloid differentiation, and HSCs self-renewal	[58]
	miR-22	TET2	HSCs maintenance and self-renewal	[59]
	miR-142-3p	IRF7	Development and differentiation	[60]
	miR-99 family		HSCs self-renewal	[61]
	miR-127-3p		HSCs differentiation and self-renewal	[62]
DCs subset	miR-142-3p		DCs development and DCs subset differentiation	[63,64,65]
	miR-221	p27kip1		
	miR-222			
	miR-155		DCs development	[66,67]
	miR-22	IRF8	DCs subset differentiation	[68]
	miR-21	JAG1, WNT1	DCs subset differentiation	[69]
	miR-34a			

**Table 2:** miRNAs involved in inflammatory response.

miRNA	Gene Target	Function	References
miR-155	TAB2 p27kip1 KPC1 SOCS-1 C/EBPβ	Expression of important cytokines as IL-1β, IL-12 and MHCII.	[65,66,67,75,76,77]
miR-221	KPC1 SOCS-1	Apoptosis and inflammation	[65]
miR-146	TRAF6 IRAK1	Anti-inflammatory mediator	[63,78,79]
miR-22	p38	Inflammatory process	[80]
miR-21		Low secretion of IL-6 and high IL-10 release	[81,82]
miR-34a	AXL	Maturation of inflammatory DCs through TLR ligand	[69,83]
miR-29	NOD2 ATF2	Secretion of pro-inflammatory cytokines involved in Crohn’s disease	[84]
miR-142-3p		Activation of adaptive immune response mediated by APC	[85,86,87]
miR-10	NOD2	Maintenance of intestinal homeostasis	[88,89]
miR-107		Maintenance of intestinal homeostasis Influence the immune response to microbiota	[90]
miR-223	C/EBPβ	Maintenance of homeostasis of the intestinal environment	[91]

## Abbreviations

DCs	dendritic cells
APCs	antigen-presenting cells
miRNA	microRNA
cDCs	conventional dendritic cells
pDCs	plasmocytoid dendritic cells
moDCs	monocytes-derived DCs
HLA	human leukocyte antigen
MHC	major histocompatibility complex
Ag	antigen
LN	lymph nodes
TLRs	Toll-like receptors
IFN	interferon
Th	T helper cells
InfDCs	inflammatory DCs
HSCs	hematopoietic stem cells
LMPP	lymphoid primed multipotent progenitor
MLP	multipotent lymphoid progenitors
GMDPs	granulocyte macrophage DC progenitor
PAMP	pathogen-associated molecular pattern
DAMP	danger-associated molecular pattern
PRRs	pattern-recognition receptors
DC-LAMP	membrane protein associated with lysosome
IL	interleukin
mRNAs	messenger RNA
3’UTR	3’-untranslated regions
Pri-miRNAs	primary miRNAs
DGCR8	DiGeorge syndrome critical region gene 8
Pre-miRNAs	precursor miRNAs
RISC	RNA-induced silencing complex
AGO	argonaute proteins
TNRC6	trinucleotide repeat-containing 6 proteins
ABCG2	ATP-binding cassette subfamily G member 2
BMF	Bcl2-modifying factor
KLF13	Kruppel-like factor 13
Dnmt3a	DNA methyltransferase 3a
BAK1	BCL2 antagonist/killer 1
TET2	ten-eleven-translocation 2
IRF	interferon regulatory factor
GM-CSF	granulocyte macrophage colony stimulating factor
M-CSF	macrophage colony stimulating factor
FLT3L	Fms-like tyrosine kinase-3 ligand
EIF2C3	eukaryotic translation initiation factor 2C, 3
CAMTA1	calmodulin-binding transcription activator 1
JAG	jagged
WNT	wingless (wg) and Int-1
TAB2	TGF-Beta activated kinase 1 (MAP3K7)-binding protein 2
SOCS	suppressor of cytokine signalling
C/EBPβ	CCAAT/enhancer-binding protein beta
TRAF6	TNF receptor-associated factor 6
IRAK1	interleukin-1 receptor-associated kinase 1
NOD2	nucleotide-binding oligomerization domain-containing protein 2
ATF2	activating transcription factor 2
BMDCs	bone marrow-derived DCs
LPS	lipopolysaccharides
TNF-α	tumor necrosis factor alpha
tDCs	tolerogenic DCs
aDCs	activated DCs
NF-kB	nuclear factor kappa-light-chain-enhancer of activated B cells
SLE	systemic lupus erythematosus
IBD	inflammatory bowel disease
CRC	colon-rectal cancer
MAPK	mitogen-activated protein kinase
